# Application of scanning acoustic microscopy for evaluation of MMP activation in multiple cancer cell lines with a smart probe

**DOI:** 10.55730/1300-0152.2652

**Published:** 2023-06-05

**Authors:** Hasan Ozan OTAŞ, Nasire ULUÇ, İrem DEMİRKAN, Aylin ALKAN, Açelya YILMAZER, Seda YAŞA, Davod KHALAFKHANY, Nesrin ÖZÖREN, Mehmet Burçin ÜNLÜ

**Affiliations:** 1 Department of Molecular Biology and Genetics, Faculty of Arts and Sciences, Boğaziçi University, İstanbul, Turkey; 2 Department of Physics, Faculty of Arts and Sciences, Boğaziçi University, İstanbul, Turkey; 3 Physics Research and Teaching Group, Middle East Technical University, Northern Cyprus Campus, Mersin 10, Turkey; 4 Department of Biomedical Engineering, Faculty of Engineering and Natural Sciences, Bahçeşehir University, İstanbul, Turkey; 5 Center for Life Sciences and Technologies, Boğaziçi University, İstanbul, Turkey

**Keywords:** Matrix metalloproteinase (MMP), cancer imaging, scanning acoustic microscope (SAM), acoustic impedance, MMPSense 680, smart probe

## Abstract

**Background/aim:**

Matrix metalloproteinases (MMPs) play an important role in the evaluation of many cancer types; however, the detection usually presents a challenge. Further assays for a better understanding of the fundamental roles of MMPs in pathophysiology are still needed. We aimed to use an activatable probe in scanning acoustic microscopy (SAM) to evaluate acoustically if the probe can aid the visualization of the effects of in vitro MMP activity.

**Materials and methods:**

We applied scanning acoustic impedance microscopy to obtain acoustic impedance maps of the cell line models of HT1080, THP-1, and SK-MEL-28 with and without MMPSense 680 probe incubation. We visually validated our results using confocal laser scanning microscopy imaging. We further analyzed the effects of MMPSense 680 probe on cell viabilities to eliminate any artifacts.

**Results:**

This is the first study presenting the applicability of SAM in the acoustical evaluation of MMPSense 680 probe cleavage in a cellular medium through acoustic impedance measurements. We proposed that SAM measurement with the activatable probe can be used as an effective tool for studying the acoustical variations of MMP activities in cell lines. As a result, we detected MMPSense 680 probe cleavage in HT1080 human fibrosarcoma cell line.

**Conclusion:**

We showed that SAM with the smart probe can detect proteolytic activity using MMPSense 680 in in vitro HT1080 cell line by acoustic impedance measurements. SAM could be proposed as an alternative tool leading a novel way for a better understanding of the roles of MMPs in cancer progression before clinical settings.

## 1. Introduction

Matrix metalloproteinases (MMPs) are a family of enzymes that specifically target the molecules of the extracellular matrix (ECM). MMPs are responsible for critical physiological activities like wound healing, apoptosis, cell migration, angiogenesis, ovulation, embryonic development in healthy cells; however, they have pivotal roles on invasion and metastasis of cancer cells. Proangiogenic MMPs degrade structural components of the ECM and have upregulated expression in several cancer types correlating with the stage, invasiveness, metastatic properties, and poor prognosis (Kessenbrock et al., 2010). Recent studies showed intracellular localization and functions of several MMPs potentially having roles in cleavage of intracellular substrates, signal transduction as well as disease pathogenesis such as cardiovascular disease, kidney disease, inflammation, and cancer development and progression (Jobin et al., 2017; Bassiouni et al., 2021). Detection and imaging of MMPs are essential for various therapeutic techniques to suppress dysregulated production of MMPs in cancer progression (Khalid and Javaid, 2016).

Fluorescence, bioluminescence, confocal and photoacoustic microscopy (PAM) have been used as noninvasive imaging modalities to visualize smart probes which are specifically designed and synthesized to be activated by MMPs (Levi et al., 2010; Xie et al., 2012; Lohela et al., 2014). These probes are based on quenched fluorophores on peptides (Fernández and Vendrell, 2016). These peptides are substrates of various MMPs and emit near-infrared fluorescence (NIRF) signal upon proteolytic cleavage (Massoud and Gambhir, 2003; Figueiredo et al., 2018). In a recent study, MMPSense 680 which is an activatable fluorescent probe was used to monitor the proteolytic activity of several MMPs (mainly 2, 9, 12, and 13) in the tumor microenvironment in real time with the confocal microscopy (Lohela et al., 2014). This study showed that the use of smart probes contributes to the advancement of research in anticancer therapies (Zhu et al., 2011; Lohela et al., 2014). Moreover, protease-activatable probe (ProSense 680) which can visualize tumor tissue via activation by proteolytic enzymes emitting NIRF signal (IRDye 800CW 2-DG) was used for the detection of tumors and metastases in various experimental models with a linear correlation between the fluorescence imaging (FLI) and bioluminescence imaging (BLI) (Xie et al., 2012). In the field of photoacoustic imaging, Levi et al. (2010) designed an activatable photoacoustic probe, which is sensitive to enzymatic cleavage by MMP-2. They detected an optoacoustic signal coming from a cell culture incubated with an activatable optoacoustic probe using absorbance-based modality. Razansky et al. (2011) employed MMPSense 680 probe and achieved a high-resolution mapping of MMP activities in the vulnerable plaque of intact human carotid specimens by tuning multispectral optoacoustic tomography (MSOT) wavelengths to activation-dependent absorption changes of the molecules.

Although the critical roles of MMPs in cancer progression have been indicated by variety of imaging tools under in vitro and in vivo conditions (Levi et al., 2010; Xie et al., 2012; Lohela et al., 2014), physical location, the time frame of MMP enzymatic activity, and MMP levels are still unknown for most cancer types. The introduction of a standard optimization method for a well-designed MMP sense probe, which can integrate dual or multiple imaging tools, is also still under investigation. Such shortcomings indicate a need for an alternative imaging technique that should be administered to overcome the current limitations by exploring the activities of MMPs quantitatively around the microenvironment of living cells.

Scanning acoustic microscopy (SAM) is an easy-to-perform imaging technique in which high frequency (>80 MHz) ultrasound is applied to quantify the variations in the reflections of sound waves interacting with cell populations and tissues. The resulting divergences can be used to get knowledge about acoustical characteristics of intracellular compartments (cytoskeleton, cytoplasm, and nucleus) or tissues. SAM can display the divergences as two-dimensional (2D) distributions of numerous parameters such as acoustic impedance, sound velocity, acoustic attenuation, and thickness (Wickramasinghe, 1983). It has also been demonstrated that acoustical properties contribute significantly to the investigation of physiological and pathological processes in the cell populations (Ingber, 2002; Bao and Suresh, 2003; Weiss et al., 2007). Considering this information, vast number of investigations have reported the potential usage of SAM to reflect the acoustomechanical properties of the biological surroundings (Strohm et al., 2010; Kobayashi et al., 2014; Fadhel et al., 2015; Miura and Yamamoto, 2015; Pasternak et al., 2015; Ito et al., 2017; Soon et al., 2017; Rahayu et al., 2018). Hence, SAM can be proposed as a complementary imaging system to currently available noninvasive methods.

In the current study, we aimed to combine the usage of MMPSense 680 probe with SAM measurements and to report whether the probe can be used to acoustically detect MMP activity in cancer cell lines. Our focus was to detect the presence of matrix metalloproteinases (MMPs) with the cleavage of MMPSense 680 smart probe in HT1080 fibrosarcoma cell line comparing with THP-1 (negative control for the experimental setup) and SK-MEL-28 melanocyte cell lines using 320 MHz SAM system. To verify the probe cleavage, we visualized all cell lines using confocal laser scanning microscopy. From the findings of our study, we concluded that we can acoustically detect the cleavage of the smart probe by MMPs in the cultured cells using SAM. Thus, this study hypothesized that SAM with 320 MHz ultrasonic transducer could be used to detect variations resulting from in vitro cleavage of MMPSense 680 probe in HT1080 human fibrosarcoma cell line by acoustic impedance measurements. Considering the capability of SAM in living cell monitoring, such preclinical investigations by SAM in cultured cells may play an essential role in understanding the underlying mechanism and the roles of MMPs in the tumor progression and angiogenesis. Therefore, in the future, SAM imaging with smart probe can be used as a complementary tool for a better understanding of the roles of MMPs in cancer progression in preclinical settings.

## 2. Materials and methods

### 2.1. Cell culture

Human fibrosarcoma cell line HT1080 (HT1080 xeno ATCC CRL-12011) and human melanoma cell line SK-MEL-28 (kindly provided by Prof. Maria Soengas - Centro Nacional de Investigaciones Oncologicas (CNIO)) were cultured in Dulbecco’s Modified Eagle Medium (DMEM) (Gibco BRL, Grand Island, NY, USA), human monocytic cell line THP-1 (kindly provided by Prof. Ahmet Gül - İstanbul University) was cultured in Roswell Park Memorial Institute (RPMI) (Gibco BRL, Grand Island, NY, USA) both supplemented with 10% fetal bovine serum (FBS). 1% MEM Non-Essential Amino Acids solution and 1% Penicillin-Streptomycin antibiotics (Gibco BRL, Grand Island, NY, USA) were added into the cell culture media to increase the cell growth and viability and to prevent contamination. For maintenance, cells were cultured in 75-cm^2^ tissue culture flasks with 15 mL of complete growth media and incubated at 37 °C in 5% CO_2_ environment. Adherent cells were passaged to keep standard confluency using 0.05% trypsin with EDTA for every 2 days. All cell lines had a passage number less than 10 and were routinely tested for mycoplasma contamination. BSL-2 type biosafety cabinets have been used with standard inspections securing the safety.

### 2.2. Cell viability assay

Measurement of cell viability was done using XTT assay (Roche Diagnostics GmbH, Germany) which depends on biochemical reduction of colorless compound to bright orange formazan derivative by enzymes derived from mitochondria of dying cells. 1 × 10^5^ cells were seeded with 100 μL of cell media into per well of 96-well plate to obtain triplicate sets per condition (control, MMPSense 680 incubation and DMSO groups). The plate was incubated at 37 °C in 5% CO_2_ environment for 16 h. The control group was incubated without addition of any compound. The MMPSense 680 group was incubated with 0.4 μM MMPSense 680 probe for 4 h. DMSO group was incubated with 10% DMSO compound for the same time. Media of the control cells were equilibrated to standardize the final volumes to other two groups. After 4 h of MMPSense 680 incubation, XTT mixture were freshly prepared by thawing XTT labeling reagent and XTT electron coupling reagent at 37 °C in water bath. Next, 5 mL of XTT labeling reagent was mixed with 0.1 mL of electron coupling reagent for one well of 96-well plate to perform the assay according to the manufactures’ instructions. XTT mixture was added into each well and incubated for 4 h at 37 °C in 5% CO_2_ environment. Spectrophotometric absorbances of the wells were measured at wavelengths of 450 nm and 600 nm separately using a microplate reader (VersaMax, Molecular Devices, USA). Absorbance values of sample at 600 nm and blank medium at 450 nm were subtracted from the absorbance value of sample at 450 nm to eliminate all nonspecific absorbances from the measurement. Analysis of XTT cell viability assay was done using the Kruskal–Wallis test. p-values less than 0.05 were considered appropriate for statistical significance.

### 2.3. Confocal laser scanning microscopy imaging

**Figure 1:**
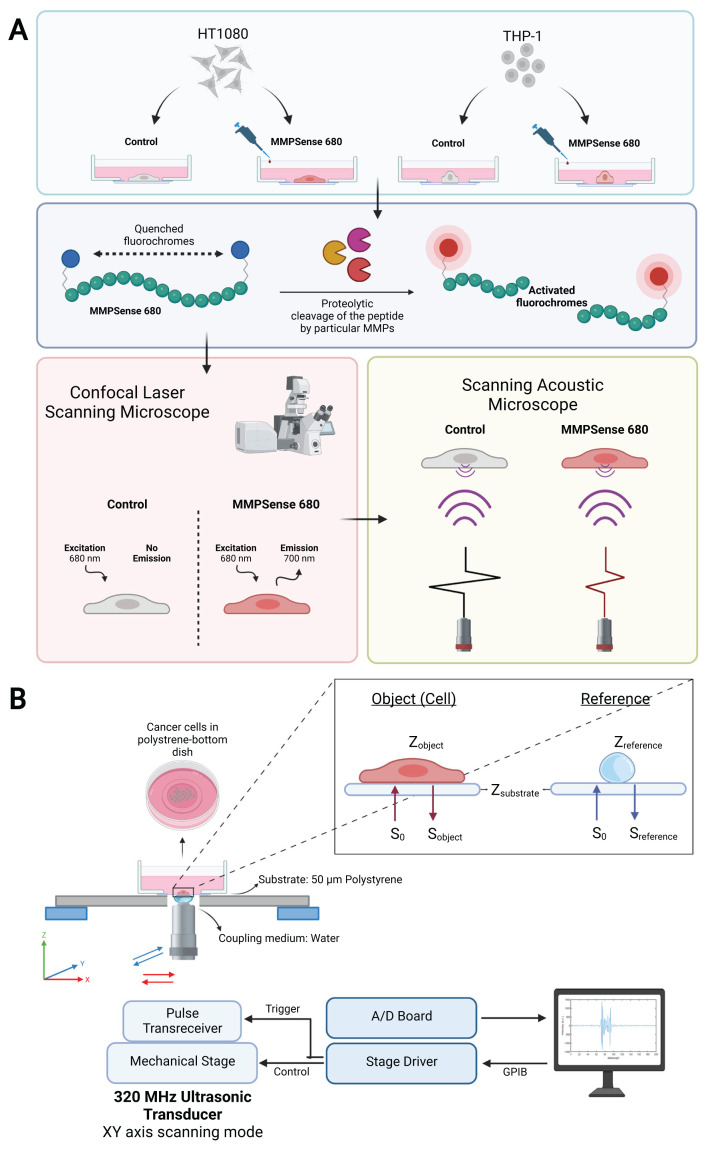
A schematic illustration (created with BioRender.com) of (A) Experimental design showing the conditions of control and MMPSense 680 incubation with the cell lines, MMPSense 680 activation by proteolytic cleavage by MMPs, confocal laser scanning microscopy imaging setup and scanning acoustic microscopy imaging setup (B) Detailed experimental setup for the scanning acoustic impedance imaging.

Cells were observed and visualized under confocal laser scanning microscope (TCS SP8 HC PL APO 40x/1.10 W, Leica). Figure 1A (created with BioRender) shows the experimental setup of cell culture along with the working principle of MMPSense 680 probe and imaging modalities. For confocal microscopy, cells were seeded onto 4-cm diameter 50-μm thick sterile polystyrene-bottom petri dishes with a cell density ranging from 2.5 × 10^5^ to 3 × 10^5^ cells/cm^2^ with 2 mL of media. For MMPSense 680 incubation, 15 μL of stock MMPSense 680 probe whose stock concentration is 20 nmol/1.5 mL was taken and transferred into cell culture media to complete totally 500 μL. In final concentration, 0.4 μM MMPSense 680 probe was added onto the cells and incubated for 4 h. The cells were then washed two times with 1X PBS to avoid nonspecific emission signals and further nonspecific impedance changes coming from inactive MMPSense 680 probe accumulations. For nucleus staining, the cells were fixed in 4% paraformaldehyde solution for 5 min and the staining was performed with 5 μg/mg DAPI solution for 15 min.

### 2.4. Scanning acoustic impedance microscopy imaging

To acoustically detect the variations between control and MMPSense 680 incubation groups of HT1080, THP-1, and SK-MEL-28 cells, we used SAM system (AMS-50SI) supplied by Honda Electronics (Toyohashi, Japan). Figure 1B (created with BioRender) displays the cartoon workflow of the SAM system and the beam propagation in the acoustic impedance measurement mode for the quantification of MMPSense 680 activation within the previously defined cells. In acoustic impedance mode shown in Figure 1B, the ultrasonic wave penetrates through polystyrene substrate and reflects at the substrate-target interface. The signals used to generate the acoustic impedance images occur at the location of the interface between the polystyrene substrate and the target. The surface of the target captures the ultrasound pulse after traveling through a polystyrene substrate having a finite attenuation. The two-dimensional distribution of acoustic impedance is acquired by mechanically scanning the ultrasonic transducer keeping the focal point on the rear surface of the substrate (Gunawan et al., 2015; Rahayu et al., 2018; Demirkan et al., 2019). In this research, SAM system, in which an ultrasonic transducer consisting of a flat ZnO thin film, an operating central frequency of 320 MHz, and a focal length of 0.5 mm with a lateral resolution of 4.7 μm was employed to image variations in the cell lines. Acoustic pulse wave for 320 MHz is variable over the range of 200–400 MHz (Soon et al., 2017). The transducer was chosen since individual cells can be clearly resolved laterally at this central frequency in a culture medium. This central frequency has stronger sensitivity in the vicinity of substrate offering proper acoustic impedance data from the sample surface. Depth penetration reduces compared to low-frequency sound waves (<80 MHz) (Bushberg, 2003). For the acoustic impedance measurement mode, the ultrasonic transducer was located under the target stage. Distilled water was chosen as a coupling liquid between the transducer and the substrate to improve acoustic impedance matching. To mount the cells on the stage, we seeded a monolayer cell to the 4-cm diameter polystyrene-bottom petri dish having a thickness of 50 μm. For each experiment, 50 μm petri dish containing cultured cells was scanned along the XY axes to sequentially penetrate all points in the field of view (FOV). This yields two dimensional (2D) color-coded acoustic impedance images. SAM functions through directing focused sound waves from an ultrasound transducer to an imaging area of the object attached to a substrate.

In this study, an ultrasonic pulse wave generated by the transducer was focused on the interface between the 50 μm polystyrene substrate and the target cells by a spherical lens. Subsequently, the sound wave which was emitted by the transducer passed through or hit the target cells and was reflected onto the surface of the target or the substrate. It was then rebounded to the detector, which was coincident with the ultrasonic transducer. Afterwards, the reflection from the dish surface was converted into a 2D image of reflection intensity and produced 2D color-coded acoustic impedance images. For each experiment, we selected the field of view to be 0.3 mm × 0.3 mm covered by a 300 × 300 scanning points. 2D scanning of the image area was performed in 1 μm scan steps. Eight pulse echo sequences with 300 scanning points were averaged for each petri dish including target cells to reduce random noise. For the image area, the total scanning time took about 2 to 3 min. Top right zoomed-in inset of Figure 1B illustrates acoustic impedance measurement mode of SAM. The target is the cell culture and the signal for the cell culture is as in the following equation (1):


$$S_{target}=\frac{\left(Z_{target}-Z_{substrate}\right)}{Z_{target}+Z_{substrate}}S_{0},$$

in which S_0_ is the signal yielded by 320MHz ultrasound transducer, Z_target_ is the acoustic impedance of the target cells, and Z_substrate_ is the acoustic impedance of the substrate material. The acoustic impedance of the cell culture is figured out by the signal reflected from the target which is then compared with that reflected from the reference. The reflected signal from the reference is as in the following equation (2):


$$S_{reference}=\frac{\left(Z_{reference}-Z_{substrate}\right)}{\left(Z_{reference}+Z_{substrate}\right)}S_{0},$$

where Z_reference_ is the acoustic impedance of the reference material. Throughout the measurement, the transmitted signal from the ultrasonic transducer is assumed constant; therefore, the acoustic impedance of the target is given as in the following equation (3):


$$\begin{array}{l} Z_{target}=\frac{1-\frac{S_{target}}{S_{0}}}{1+\frac{S_{target}}{S_{0}}}Z_{substrate}\\ =\frac{1-\frac{S_{target}\left(Z_{substrate}-Z_{reference}\right)}{S_{reference}\left(Z_{substrate}+Z_{reference}\right)}}{1+\frac{S_{target}\left(Z_{substrate}-Z_{reference}\right)}{S_{reference}\left(Z_{substrate}+Z_{reference}\right)}}Z_{substrate} \end{array}$$

where Z_reference_ and Z_substrate_ are the acoustic impedance of the reference material and substrate, respectively. For the reference material, cell culture medium (Z_reference_ = 1.52 MRayl) was employed since it was assumed to be acoustically similar to water. Polystyrene-bottom dish (Z_substrate_ = 2.37 MRayl) was selected as the substrate. Prior to the observation of the cells, the system was calibrated through a signal recorded from the polystyrene-bottom dish only without the cells. Within the above equation, reflections from the target S_target_ and the reference S_reference_ are both directly measurable signals, whereas transmitted signal from the transducer S_0_ is not. The acoustic impedance equation for the cells equation 3 was basically corrected through considering the focused sound field (Strohm et al., 2010; Kobayashi et al., 2014; Fadhel et al., 2015; Gunawan et al., 2015; Miura and Yamamoto, 2015; Pasternak et al., 2015; Ito et al., 2017; Soon et al., 2017; Rahayu et al., 2018; Demirkan et al., 2019).

### 2.5. Data and statistical analysis

In this investigation, HT1080, SK-MEL-28, and THP-1 cell lines were assessed by GraphPad software (http://www.graphpad.com/scientific-software/instat). We arbitrary selected single cells from three separate sets of cells prepared under the same conditions for each cell line. Among them, significances of differences were analyzed for control and MMPSense 680 incubation groups (n = 25 cells). Therefore, the data from three separate measurements were unified into average ± SDs of 25 single cells for each cell line. At this time, 25 single cells without the MMPSense 680 incubation and 25 single cells with the MMPSense 680 probe incubation were analyzed. Image analysis was performed by ImageJ software (NIH: https://imagej.nih.gov/ij/) and image processing toolbox of MATLAB (MathWorks, Inc.). The cells were distinguished from the cell medium using a threshold. The threshold was employed in all acoustic impedance distributions, to suppress the impedance of the background without removing the cells from the field of view. All cell lines had the same characteristics media. Thus, the threshold was determined using the mean value of the first minimum from the smoothened histogram of the acoustic impedance images, and it was determined as 1.520 MRayl for our study. After determining the threshold, the verge of the cells was marked and analyzed as a whole scattering source by region of the interest analysis tool (Fadhel et al., 2015). Moreover, acoustic wave forms for each cell were analyzed to understand the reason of possible artifacts and determine the second threshold to remove artifacts. Quantitative results for both acoustic impedance and confocal microscopy measurements were conducted using t-test followed by the Mann–Whitney U analysis to determine statistically significant differences between control and MMPSense 680 incubation groups of HT1080, THP-1, and SK-MEL-28 cells.

## 3. Results

### 3.1. Acoustic impedance value of MMPSense 680 probe was detected

**Figure 2:**
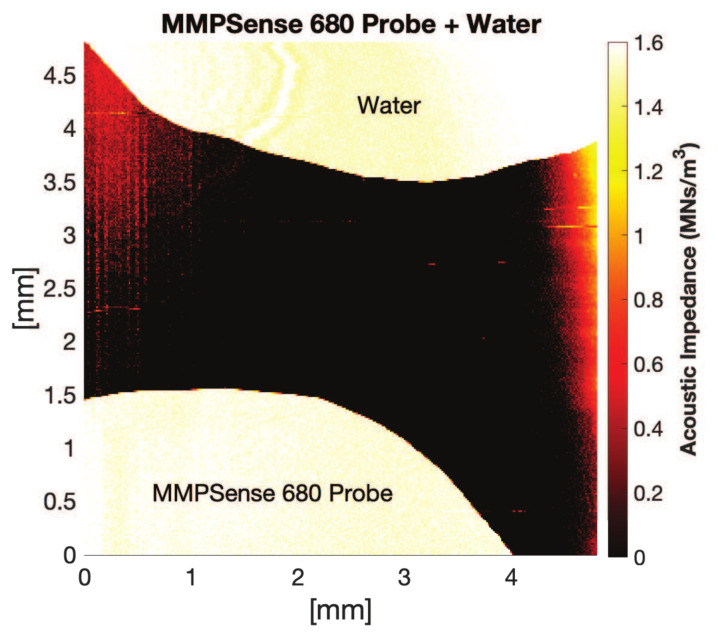
Acoustic impedance map of MMPSense 680 probe. The top side: Acoustic impedance of the water (1.520 MN×s/m^3^). The bottom side: Acoustic impedance of the MMPSense 680 probe (1.533 MN×s/m^3^) (1 MN×s/m^3^ = 1 MRayl).

We optimized the detectable concentration of the probe by comparing the acoustic impedance of distilled water with SAM. The mean acoustic impedance of the noncleaved MMPSense 680 probe (0.4 μM) was calculated as 1.533 MRayl, shown in Figure 2. The acoustic impedance of the distilled water and MMPSense 680 probe were shown on the top side and the bottom side of the figure, respectively.

### 3.2. Cleavage of MMPSense 680 probe resulted a significant decrease of acoustic impedance in HT1080 fibrosarcoma cells

**Figure 3:**
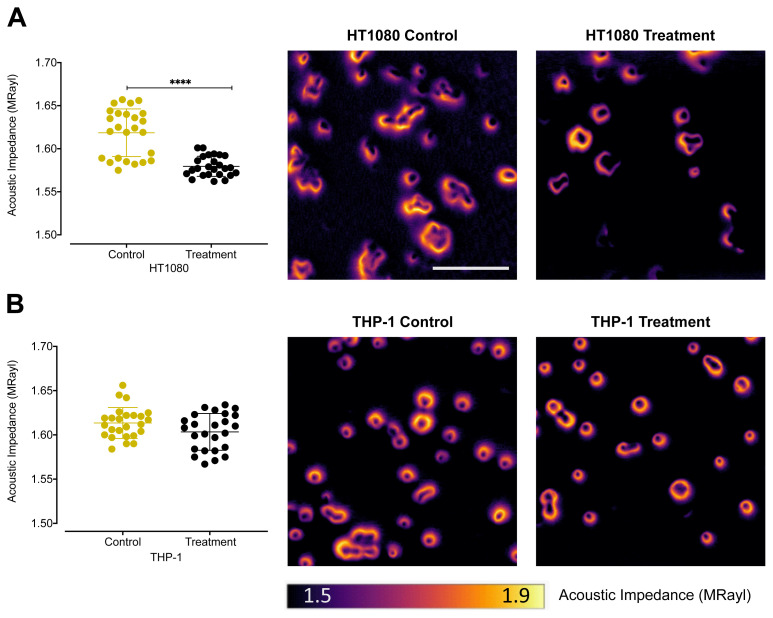
Acoustic impedance values (MRayl) and acoustic impedance images of control and treatment (MMPSense 680 incubation) groups of (A) HT1080 and (B) THP-1 cells. Field of view is 0.3 mm × 0.3 mm with 300 × 300 scanning points (scale bar = 100 μm). Impedance values of 25 single cells were investigated for each condition. A significance of difference among the cell lines in terms of acoustic impedance values was shown through the asterisks as ****p < 0.0001.

The acoustic impedance distributions of HT1080, THP-1, and SK-MEL-28 cells with and without MMPSense 680 probe incubation were investigated using 320 MHz SAM. Acoustic impedance images and individual cell measurement plots of control and MMPSense 680 incubation groups of HT1080, THP-1, and SK-MEL-28 are shown in Figures 3A, 3B, and S1, respectively. Factual data for the average acoustic impedance values, standard deviations, and reduction in percentages for the acoustic impedance of each cell type were also summarized in Table.

Figure 3A shows the acoustic impedance values of control and probe incubation groups of HT1080 cell line. The results indicated that the acoustic impedance values of the cell line vary significantly, enabling to state a statistical difference between control and MMPSense 680 probe incubation groups. For HT1080 control group, we observed the acoustic impedance value provided by SAM (Table) as 1.638 ± 0.034 MRayl which is statistically significant (p < 0.0001) when compared with HT1080 MMPSense 680 probe incubation group whose impedance value is 1.590 ± 0.009 MRayl. The mean acoustic impedance of HT1080 decreased by 3%. THP-1 (Figure 3B) and SK-MEL-28 (Figure S1) did not show any statistically significant change in terms of acoustic impedance.

### 3.3. Cleavage of MMPSense 680 probe resulted a detectable fluorescence signal emission in HT1080 fibrosarcoma cells

**Figure 4:**
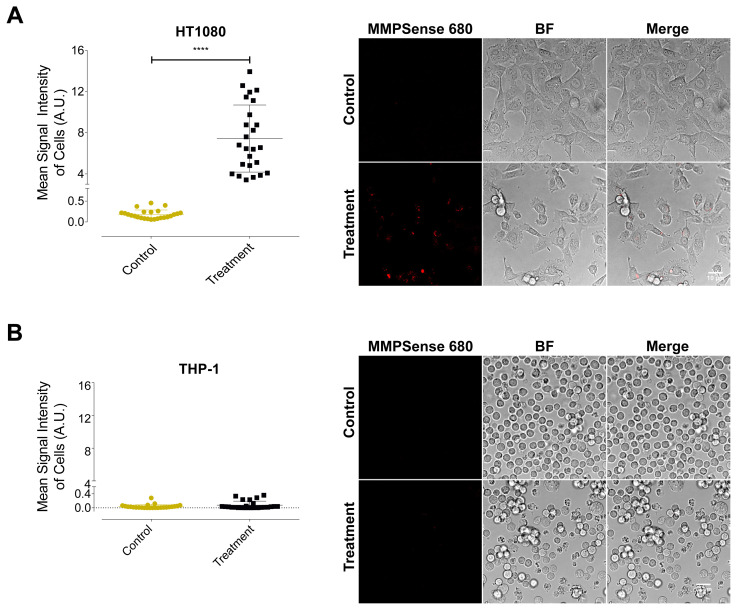
Confocal microscopy and mean intensity results of control and treatment (MMPSense 680 incubation) groups of (A) HT1080 and (B) THP-1 cells. 40X magnification with water immersion lens was used. Scale bar = 10 μm. BF = Bright Field image. *t*-test with Mann-Whitney U calculation was used (****p < 0.0001).

**Figure 5:**
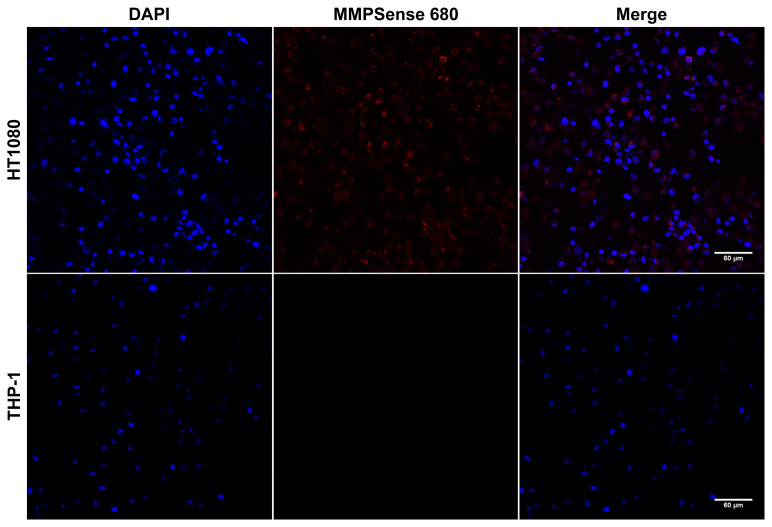
MMPSense 680 probe activation signal was detected outside of the nucleus in HT1080 cells. DAPI was used for nucleus staining after MMPSense 680 incubation in HT1080 and THP-1 cell lines. Excitation/emission wavelengths are 405/410–550 nm for DAPI, 638/643–717 nm for MMPSense 680 probe. Scale bar = 60 μm.

To visualize the activities of MMPs, HT1080, THP-1, and SK-MEL-28 cell lines were incubated with MMPSense 680 probe and images were taken using confocal laser scanning microscopy. Figure 4A illustrates the results of HT1080 cell line in which MMPSense 680 showed emission of fluorescent signal after activation by particular MMPs. Cleavage of the probe was quantified measuring mean intensity change of the emission signal between HT1080 control and MMPSense 680 incubation groups. Also, HT1080 cell line was the only group whose MMPSense 680 probe activation was visually detectable using confocal microscopy as in Figure 4A. In HT1080 cell line, the mean intensity of the fluorescence emission signal in control group was measured as 0.158 arbitrary unit (A.U.); on the other hand, the mean intensity of the MMPSense 680 incubation group displayed a significant increase to 6.552 A.U. (p < 0.0001) in HT1080 showing the highest change among the other cell lines. Nearly 41-fold increase in terms of signal intensity between control and MMPSense 680 incubation groups of HT1080 cells were observed. Mean intensity graphs of THP-1 (Figure 4B) and SK-MEL-28 (Figure S2A) showed neither a visible signal nor a significant change. According to the mean intensity changes of THP-1 monocytic cells in Figure 4B, control group showed the mean intensity value of 0.018 while the MMPSense 680 incubation group displayed the value of 0.028. Confocal laser scanning microscopy also allowed us to discriminate between cellular compartments visually staining the nuclei of HT1080, THP-1 (Figure 5), and SK-MEL-28 (Figure S2B). The merged image of the nucleus staining and visible signal emission of HT1080 cells after enzymatic cleave of the probe indicated that MMPSense 680 activation signals came from the region beneath the cell membrane as widely distributed inside of the cells. Also, the nucleus signal did not overlap with the MMPSense 680 probe activation signal which is an indicator for the intracellular localization of MMPs specific for MMPSense 680 probe. On the other hand, THP-1 and SK-MEL-28 did not show a visible intracellular signal from probe upon the MMPSense 680 incubation.

To further evaluate whether MMPSense 680 influences cell thickness, we obtained z-stack images of the control and MMPSense 680 incubation groups of HT1080 cell line using confocal microscope. The z-sections between the most upper and the most lower layers of the DAPI channel allowed us to construct z-axis plots selecting ten cells per group, randomly. Full width at half maximum (FWHM) of the reconstructed profiles in z direction allowed us to show a comparative analysis between control and MMPSense 680 groups. Our confocal analysis shown in Figure S3 indicated no significant difference (p = 0.56) between average nucleus thicknesses of the control and MMPSense 680 incubation groups of the cells indicating that MMPSense 680 incubation does not affect the cell thickness; thus, the acoustic signals obtained from the MMPSense 680 incubation groups are without any artifacts caused by changes in thickness.

### 3.4. MMPSense 680 probe incubation did not cause a destructive effect on cell viabilities

**Figure 6:**
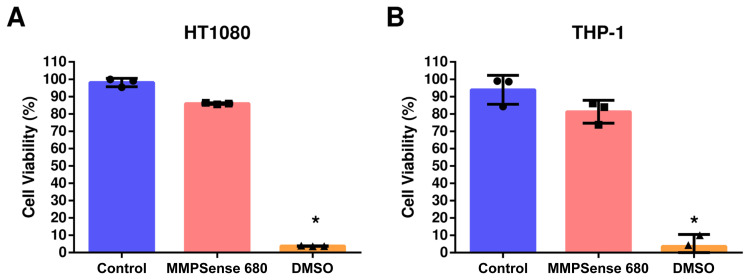
XTT cell viability assay. Control, MMPSense 680 incubation and DMSO groups were compared in (A) HT1080 and (B) THP-1 cell lines. Kruskal–Wallis one-way analysis of variance has been done (*p < 0.05).

To investigate whether MMPSense 680 probe has any detrimental effects on cell viabilities, we used XTT cell viability assay including control, MMPSense 680 probe incubation and DMSO (killing control) groups. Cell viability results of HT1080, THP-1, and SK-MEL-28 cells are shown in Figures 6A, 6B, and S4, respectively. While control group of HT1080 showed 98.09% viability, MMPSense 680 probe incubation group showed 85.46% cell viability. DMSO killing-control showed decreased cell viability to 3.63% which was statistically significant (p < 0.05) when compared with control and MMPSense 680 incubation groups. THP-1 control group cells showed 93.91% cell viability when compared with MMPSense 680 probe incubation which yielded 81.25% cell viability. DMSO killing-control significantly (p < 0.05) decreased the cell viability to 3.64% comparing control and incubation groups as shown in Figure 6B. Effects of MMPSense 680 incubation on SK-MEL-28 cells are shown in Figure S4 where the control group had 95.73%, MMPSense 680 incubation group had 78.81% and DMSO control group had 1.44% cell viabilities. Cell viability in DMSO group of SK-MEL-28 cells were significantly (p < 0.05) decreased when compared with control and MMPSense 680 incubation groups.

## 4. Discussion

MMPSense 680 is an activatable probe whose structure is changed upon activation especially by MMP-2,-3,-9,-13 (Stanton et al., 1998; Razansky et al., 2012; Kim et al., 2014). Thus, the probe was chosen to detect the proteolytic dynamics of the several MMPs in cancerous cells. All the three cell lines were treated with 0.4 μM MMPSense 680 activatable probe. After 4 hours of exposure to the probe, we visualized the same cells using both confocal laser scanning microscopy and SAM. The influence of MMPSense 680 probe on the HT1080 cells was anticipated to be significant than the other cell lines used in the study in agreement with previous studies reported that HT1080 cells produce considerable amount of MMPs (Stanton et al., 1998; Kim et al., 2014). We validated our hypothesis using confocal microscopy and showed the visible signal emission resulted by activated MMPSense 680 probe. Moreover, we measured acoustic impedance value of the enzymatically undigested MMPSense 680 probe to evaluate the effective value in the acoustic impedance range of the cells as shown in Figure 2. As expected, the MMP probe was cleaved in the presence of MMPs, which led to the most notable reduction in terms of acoustic impedance values in HT1080 fibrosarcoma cells as shown in Figure 3A. The mean acoustic impedance value of 25 single human fibrosarcoma cells decreased by about 3% when MMPSense 680 probe was activated within the cells. We also reported that, incubation of MMPSense 680 probe for 4 hours did not cause any significant change on cell viabilities (Figure 6A) and their thicknesses (Figure S3) between control and MMPSense 680 incubation groups.

In our study, we proved that we can acoustically detect the cleavage of this smart probe by the MMPs in human fibrosarcoma cells using SAM however, due to the acoustic impedance measurement mode and the frequency we used, we had imaging limitations which need to be addressed. A high-frequency ultrasound beam (> 80 MHz), which has a small wavelength, offers superior image resolution laterally and detailed microstructure observation than a low-frequency beam. On the other hand, at high frequencies, the beam penetration depth reduces. 320 MHz SAM has stronger sensitivity in the vicinity of polystyrene substrate to visualize elastic properties and distinguish a single cell in a culture medium, yet it does not give sufficient acoustic impedance data in-depth. By using acoustic data waveforms, the reduction in the acoustic impedance at the centre of the cell is supposed to be an artifact. This derives from the fact that the interpretation is done by simply comparing the intensity of the whole wave forms (in root mean square). As a future work, the cross-sectional acoustic impedance would be possible to enhance if the waveform has been taken with a high sampling rate (like 0.2 ns), but the technique is still in progress (Nagaoka et al., 2019; Prastika et al. 2020). In this regard, we eliminated the artifacts using a threshold. Therefore, reduced values in the average acoustic impedance of the treated cell groups can be reasoned by cleavage of the probe in HT1080 cells which are known to have high MMP activity. In accordance with the results, we inferred that acoustic impedance measurements using SAM can pave the way as a promising technique to detect probe cleavage in cells having a considerable amount of MMPs.

The main purpose of this research was to take benefit of using MMPSense 680 smart probe in SAM with the advantages of non-destructive mechanisms at microscale resolution and to assess acoustically if this probe can aid the visualization of the effects of MMPs in cell lines. With this research, we showed that SAM can detect the proteolytic activity using MMPSense 680 probe in in vitro HT1080 cell line by acoustic impedance measurements. Accordingly, in future, SAM may lead a novel way for better understanding of the roles of the MMPs in cancer progression in pre-clinical settings.

## Supplementary material

Figure S1 Acoustic impedance values (MRayl) and acoustic impedance image of control and treatment (MMPSense 680 incubation) groups of SK-MEL-28 cell line. Field of view is 0.3 mm × 0.3 mm with 300 × 300 scanning points. Impedance values of 25 single cells were investigated for each condition.

Figure S2 Confocal microscopy imaging of SK-MEL-28 cell line. (A) Confocal images (right) and mean intensity results (left) of control and treatment (MMPSense 680 incubation) groups of SK-MEL-28 cells. 40× magnification with water immersion lens was used. Scale bar = 10 μm. t-test with Mann-Whitney U calculation was used. (B) DAPI nucleus staining of SK-MEL-28 cells together signal emission for MMPSense 680 after MMPSense 680 incubation for 4 hours. Excitation/emission wavelengths are 405/410–550 nm for DAPI, 638/643–717 nm for MMPSense 680. Scale bar = 60 μm.

Figure S3 Evaluation of HT1080 cell thickness using Confocal Laser Scanning Microscopy. (A) Montage of 8 frame from z-stack series of HT1080 cell line with DAPI (blue) and MMPSense 680 (red) incubation. (B) Comparison of individual cell thicknesses using FWHM method on z-axis profiles of control (left) and MMPSense 680 incubation (right) groups. (C) Comparison of cell thicknesses of randomly selected 10 cells from control and

Figure S4 XTT cell viability assay of control, MMPSense 680 and DMSO groups of SK-MEL-28 cell line. Kruskal–Wallis one-way analysis of variance test has been done (*p < 0.05).

## Figures and Tables

**Table:** Acoustic impedance values of control and MMPSense 680 probe incubation groups of the cell lines. For each group, 25 single cells were analysed regarding the result of waveform analysis to figure out the correlation of control and cleaved MMPSense 680 probe activity. (NS = Not significant)

Cell types	Control Groups (MRayl)	MMPSense 680 Activated Groups (MRayl)	Reduction (%)
**HT1080**	1.638 ± 0.034	1.590 ± 0.009	3.0
**SK-MEL-28**	1.607 ± 0.023	1.599 ± 0.017	NS
**THP-1**	1.600 ± 0.002	1.593 ± 0.019	NS

MMPSense 680 incubation groups. t-test followed by Mann-Whitney-U calculation was applied. Mean differences are found as not significant (p = 0.56).
