# Supplementary figures and images for: Application of scanning acoustic microscopy for evaluation of MMP activation in multiple cancer cell lines with a smart probe
Source: Turk J Biol. 2023 Jun 5;47(3):158–69. doi: 10.55730/1300-0152.2652 (PMC10387868; doi:10.55730/1300-0152.2652)

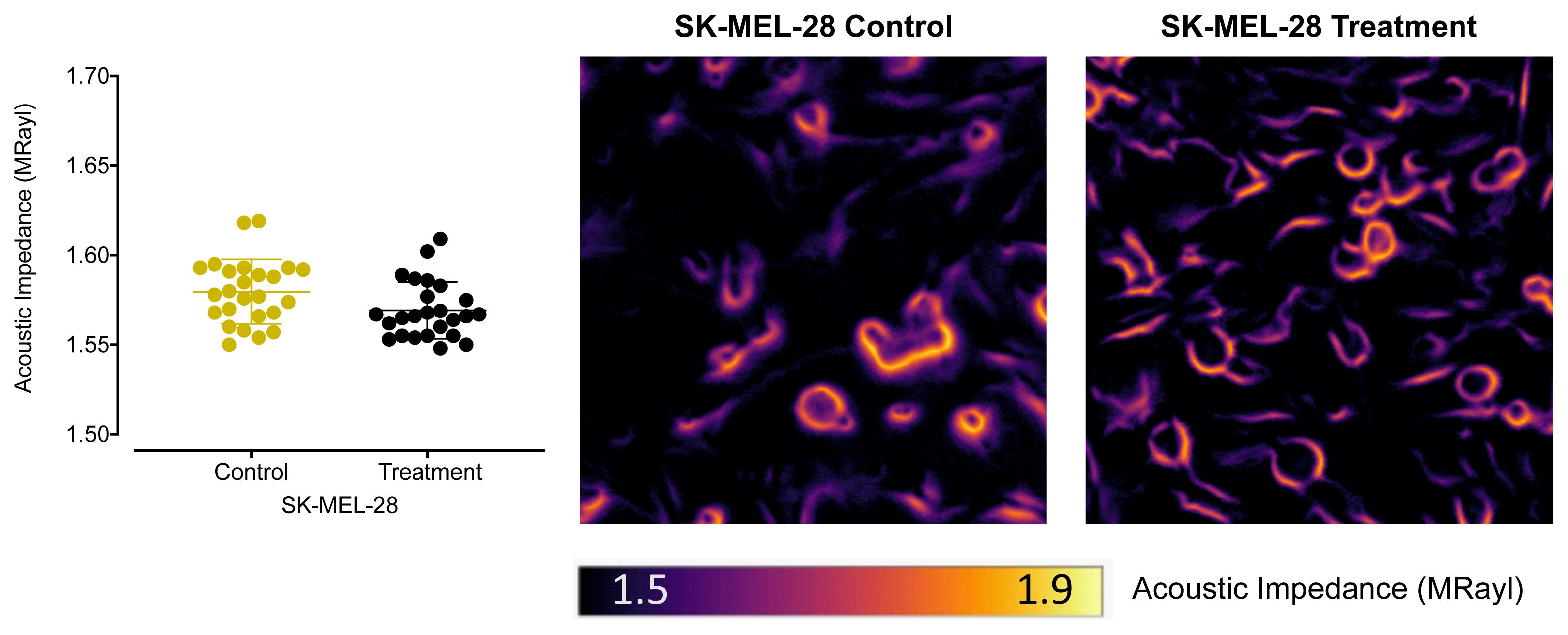

Supplement: Figure S1 — Acoustic impedance values (MRayl) and acoustic impedance image of control and treatment (MMPSense 680 incubation) groups of SK-MEL-28 cell line. Field of view is 0.3 mm × 0.3 mm with 300 × 300 scanning points. Impedance values of 25 single cells were investigated for each condition. [file turkjbiol-47-3-158s1.tif]

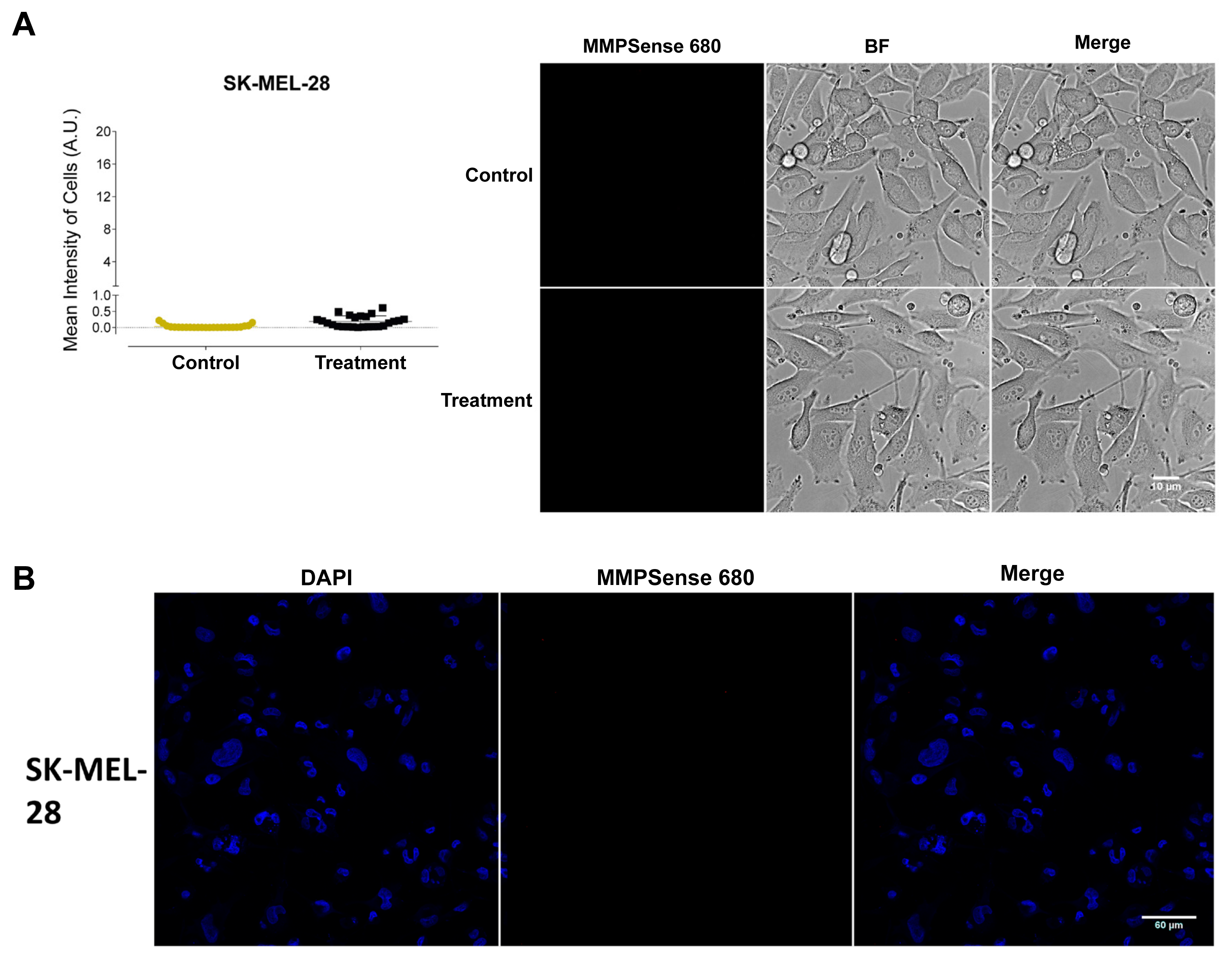

Supplement: Figure S2 — Confocal microscopy imaging of SK-MEL-28 cell line. (A) Confocal images (right) and mean intensity results (left) of control and treatment (MMPSense 680 incubation) groups of SK-MEL-28 cells. 40× magnification with water immersion lens was used. Scale bar = 10 μm. t-test with Mann-Whitney U calculation was used. (B) DAPI nucleus staining of SK-MEL-28 cells together signal emission for MMPSense 680 after MMPSense 680 incubation for 4 hours. Excitation/emission wavelengths are 405/410–550 nm for DAPI, 638/643–717 nm for MMPSense 680. Scale bar = 60 μm. [file turkjbiol-47-3-158s2.tif]

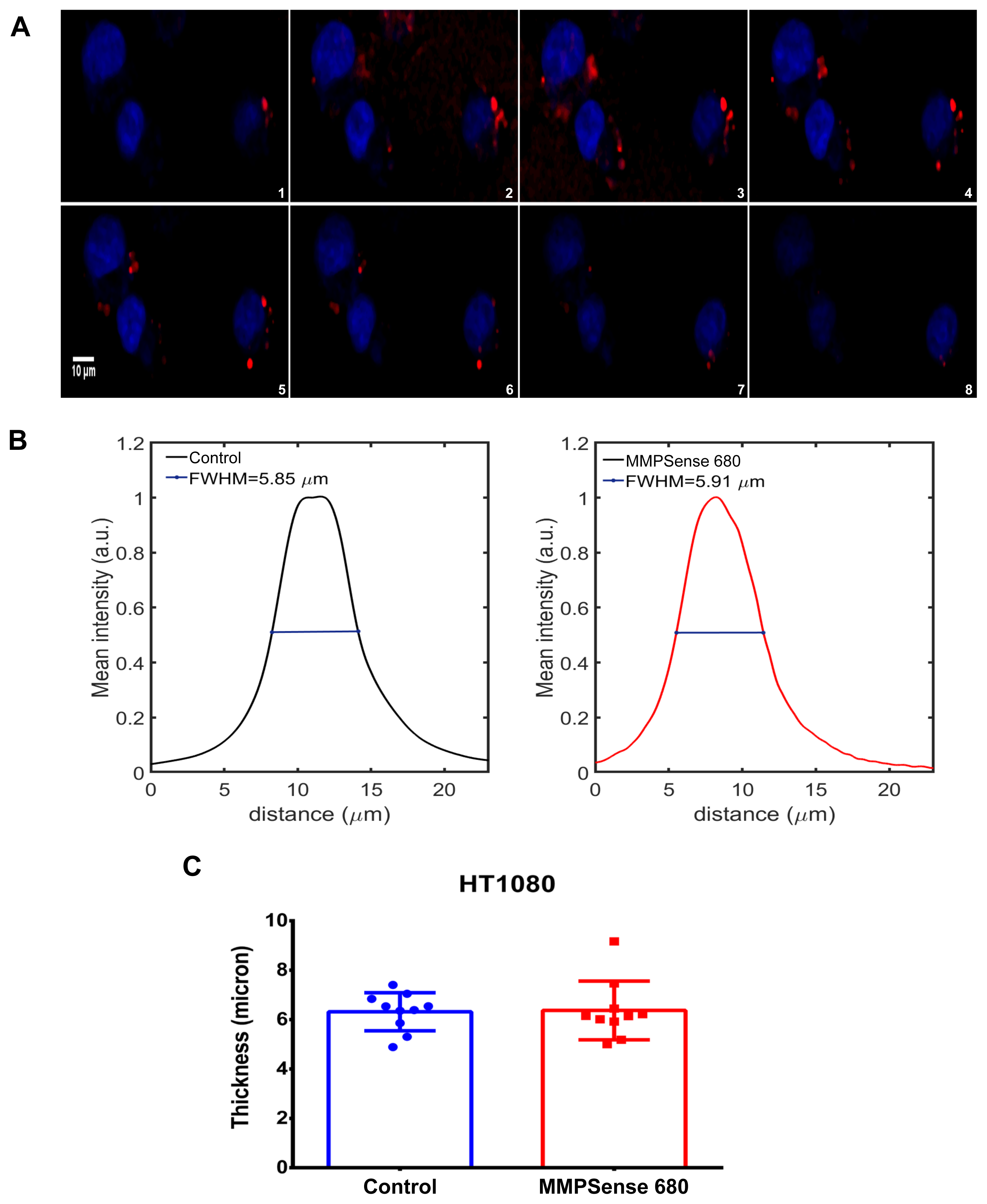

Supplement: Figure S3 — Evaluation of HT1080 cell thickness using Confocal Laser Scanning Microscopy. (A) Montage of 8 frame from z-stack series of HT1080 cell line with DAPI (blue) and MMPSense 680 (red) incubation. (B) Comparison of individual cell thicknesses using FWHM method on z-axis profiles of control (left) and MMPSense 680 incubation (right) groups. (C) Comparison of cell thicknesses of randomly selected 10 cells from control and [file turkjbiol-47-3-158s3.tif]

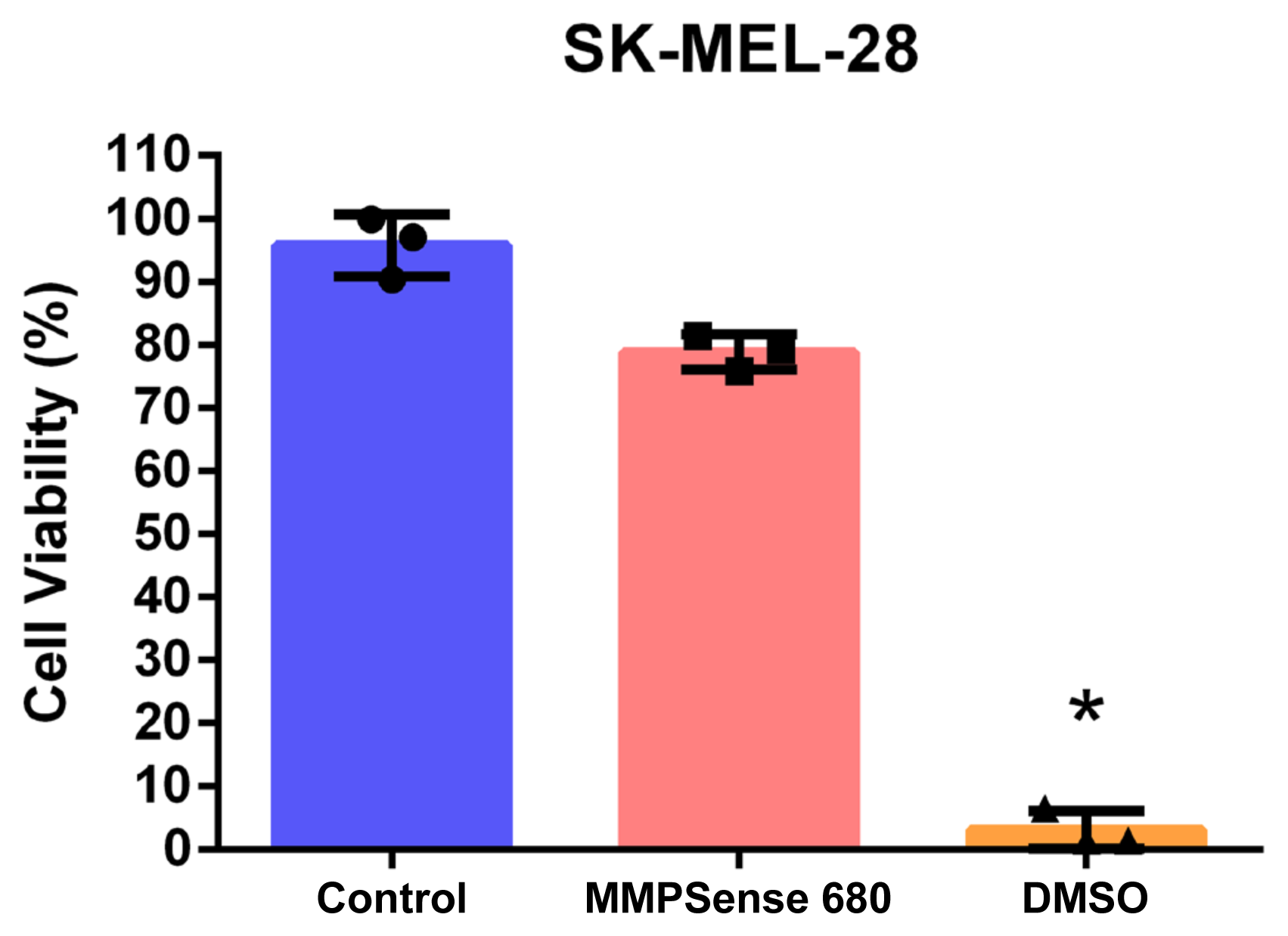

Supplement: Figure S4 — XTT cell viability assay of control, MMPSense 680 and DMSO groups of SK-MEL-28 cell line. Kruskal–Wallis one-way analysis of variance test has been done (*p < 0.05). [file turkjbiol-47-3-158s4.tif]
